# Laser-Based Door Localization for Autonomous Mobile Service Robots

**DOI:** 10.3390/s23115247

**Published:** 2023-05-31

**Authors:** Steffen Müller, Tristan Müller, Aamir Ahmed, Horst-Michael Gross

**Affiliations:** Neuroinformatics and Cognitive Robotics Lab, Technische Universität Ilmenau, 98693 Ilmenau, Germanyhorst-michael.gross@tu-ilmenau.de (H.-M.G.)

**Keywords:** laser range scan, door localization, PointNet, deep learning

## Abstract

For autonomous mobile service robots, closed doors that are in their way are restricting obstacles. In order to open doors with on-board manipulation skills, a robot needs to be able to localize the door’s key features, such as the hinge and handle, as well as the current opening angle. While there are vision-based approaches for detecting doors and handles in images, we concentrate on analyzing 2D laser range scans. This requires less computational effort, and laser-scan sensors are available on most mobile robot platforms. Therefore, we developed three different machine learning approaches and a heuristic method based on line fitting able to extract the required position data. The algorithms are compared with respect to localization accuracy with help of a dataset containing laser range scans of doors. Our LaserDoors dataset is publicly available for academic use. Pros and cons of the individual methods are discussed; basically, the machine learning methods could outperform the heuristic method, but require special training data when applied in a real application.

## 1. Introduction

Autonomous mobile service robots can be a useful aid in many applications. While there are autonomous transport systems in hospitals and industry, for many other potential operational areas, invasive installations with remote-controlled doors and elevators are not possible. Thus, robots with transportation tasks need to be able to open doors with on-board capabilities. In order to perform the required manipulations correctly, the robots need to identify and localize the key features of a door, i.e., the handle for grasping it and the hinge, which defines the pivot point for the circular opening motion. Furthermore, the exact opening angle of a door needs to be determined to derive the necessary action; a closed door, for example, needs to be unlatched first, while a partly open door only needs to be pushed open.

There are a couple of publications presenting door manipulating robots which in first instance rely on image analysis for detecting the handles and doors at all. Arduengo et al. [1], for example, used a specialized YOLO (You Only Look Once) detector trained on doors and handles. Unfortunately, these image detectors only yield 2D bounding boxes; thus, subsequent data from a depth camera are needed to extract 3D positions. Furthermore, point clouds of depth cameras are used to identify the desired door features [2,3,4,5]. Here, the approach is often an indirect one. Since identification of door handles and hinges is difficult, the plane of the door panel, which in contrast is easy to recognize, is segmented in first place. Then, the structure of the door handle results from subtracting all plane points from the region of interest crop, and the centroid of the handle points serves as the target point for a robotic grasp.

None of the publications have dealt with a systematic study of the accuracy of their door feature localization. In the first instance, the position accuracy is determined by the resolution, noise, and completeness of the point clouds, assuming that the region of interest (e.g., the handle) can be cropped correctly. Since image-based methods were successfully used to manipulate doors with robotic grippers, the localization error seems to be in a low centimeter range.

Nevertheless, image- and 3D-point-cloud-based methods also have drawbacks. We found that conventional depth cameras (either time of flight or active stereo) have difficulties to reproduce curved metallic surfaces with narrow radii, the shape of many door handles. Furthermore, bad illumination conditions and material dependent artifacts in depth data of that important region can disturb robust recognition. Additionally, during the manipulation, the gripper of the robot might obscure the view of the door handle, which limits the manipulation to offline approaches that select a target only once and execute the movement without further corrections.

An alternative to vision and depth-data-based localization is the analysis of 2D laser range scan data (LiDAR). Most of today’s robots are equipped with LiDAR sensors which are used for navigation purposes (self localization and obstacle avoidance). This suggests that these sensors can also be used for analyzing the doors and detecting the required manipulation poses indirectly. When the hinge and the endpoint of the door panel can be localized, we can compute the exact manipulation poses on the handle and the necessary arc movements based on known offsets of the handles pivot point to the door panel’s border. Furthermore, by detecting the opposite side of the door’s frame (called the lock point), the exact opening angle of the door can be derived when the hinge to lock line is compared to the line of the door panel.

Therefore, in this article, we study four different methods for localizing the door in horizontal laser-range scans taken from the robot’s perspective. First, we introduce a heuristic approach that relies on line fitting. Similar methods already exist and have been mainly used to observe the door opening angle during manipulation, rather than for defining the grasp pose at the handle. We were able to improve the method in that direction and to realize a vision-independent door-manipulating mobile robot. In addition to the heuristic method, we developed and evaluated two neural networks. These networks are able to predict the doors’ key features (hinge, lock, and corner), which together with a priory knowledge on the doors’ geometry, can be used to compute 3D poses at the handle used for manipulation. In contrast to neural network-based detection algorithms (mostly based on classification), our networks are used for the regression of exact coordinates in the 2D plane (top view). Therefore, we could evaluate the methods by means of exact distance errors of the 2D keypoints directly instead of giving only overlap-based detection accuracy. We will show that the position error could be halved by the machine learning methods.

## 2. Problem Formalization

Although a LiDAR-based approach demands significantly less computational effort compared to deep learning image-based methods, a laser range scan alone is not that informative. It is hard to identify closed doors on a flat wall without additional prior information. Furthermore, the line segments of open doors can easily be confused with other furniture.

Fortunately, for many robotic installations, the operational area is known before. Usually, mapping takes place at the beginning, where doors on the way can be registered in a database. Here, also the more sophisticated image-based methods together with depth-data analysis can be used to recognize additional data on doors, such as the opening direction and the width of the door. By showing the robot the door at different opening states, the coordinates of the door’s hinge and lock can be localized in map coordinates as well. The hinge, for example, is given as the pivot point of the arc motion of the door panel during an opening movement.

Once the initial recording has been completed, the problem during normal operation is only to identify the door’s current state (opening angle) and to localize the door’s features (handle and hinge) exactly with respect to the robot-centered coordinates. Here, the global localization error of the robot’s navigation skills must be compensated. Usually, this offset to the map coordinates is below 10 cm, but for manipulation without any feedback, this is still too much.

**Figure 1:**
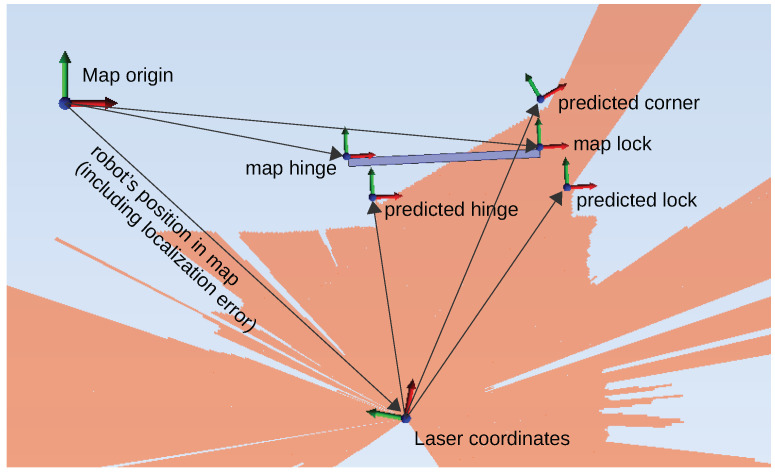
In orange, a LiDAR scan from a robot’s top view perspective in front of a door seen from outside is shown. Given the map’s hinge and lock positions, the exact positions of the hinge, lock, and corner of the door in laser coordinates have to be predicted. The deviation of the mapped hinge and lock to the actual door results from the localization error of the robot with respect to its map.

**Figure 2:**
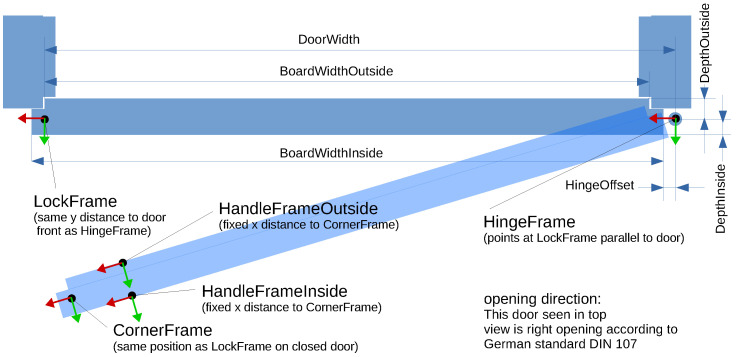
Illustration of a door’s parameters defining its appearance in a laser scan.

Therefore, our autonomous robot needs to have a database of doors in its operational area containing the following information:

- The door’s opening direction (left or right according to the German standard DIN 107);
- The door’s hinge and lock position given in map coordinates (see Figure 1);
- Parameters of the door blade geometry specifying its appearance: width inside, width outside, offset to the hinge, depth inside, and depth outside (see Figure 2);
- For manipulation, the relative positions of the door handle on the inside as well as outside to the corner of the door blade are stored.

Figure 2 illustrates these parameters.

At run time, a laser range scan r(α) is given, containing range measurements in equidistant angular steps in a horizontal plane. The exact height of the scan plane depends on the robot hardware, but due to the vertically oriented faces of walls and door, this is not critical. In application, we know a coarse position of the range scan’s origin with respect to the map coordinate system from the robot’s localization system since the LiDAR is fixed to the mobile robot. In those map coordinates, the a priory door data are also given (see the blue bar in Figure 1). The deviation in position and orientation depends on the localization method of the robot’s navigation system, and in our case, this was assumed to be limited to ±10 cm and ±10°.

The goal now is to find the exact position of a visible door’s hinge, lock, and corner of the board with respect to the range scan origin as shown in Figure 1. Since the LiDAR is fixed to the robot, the extracted position data can be transformed to robot-centered coordinates as necessary for manipulation later on.

## 3. Related Work

In the literature, there are few laser-range-scan-based methods for analyzing a door. The range scan data are often used as a supplementary cue in order to restrict other vision or 3D data-based methods for exact localization. Shi et al. [6] used the laser range data to detect possible door candidates. Those are further filtered by reasonable door widths. The main aspect of this detection algorithm was to decrease the localization error of a mobile robot, navigating through a real-world scenario, while no exact analysis of the remaining error is provided.

Endres et al. [7] segmented a line, representing the door’s panel, to calculate the current angle of a door in laser coordinates. The detected angle was then used to calculate the force for a push motion to bring the door to a desired goal angle. Again, the accuracy of the angle measurement is not given. Once the door has been observed at various angles during the motion, the hinge position was calculated as the pivot of the rotation motion. This limits the applicability for preparing the manipulation when the door is a static obstacle.

Another approach where only laser data are utilized to detect a door frame is given in [8]. This particular approach is only able to detect doors when the robot is in between the frame of an open door by analyzing distance minima heuristically. Therefore, it is of minor relevance to our scenario.

The processing of laser scans in other domains aims to detect objects such as cars or people. The focus here is the recognition of peoples’ presence rather than their exact position. Beyer et al. [9] processed the scan data by means of a Convolutional Neural Network, which classifies individual segments of a scan. Unfortunately, they only analyzed the detection accuracy, while a positive detection is defined by a ground truth label within a range of 0.5 m.

A different approach for processing the polar coordinate data of the range scan was described by Chen et al. [10]. They detected vehicles and pedestrians by painting the top view of the point clouds into a 2D image, which afterwards was analyzed by a 2D Convolutional Network from the image domain. Again, this is a detection approach with a limited spatial accuracy that is limited by the image resolution.

Since neither of the related papers directly addressed the exact localization of door keypoints and performed an objective quantitative evaluation of the localization error, we decided to create an own dataset and created a benchmark of different possible approaches that have been derived from networks for other domains.

## 4. Dataset

In order to evaluate and train different methods for door localization, we recorded a dataset of 20 different doors in public buildings of our university as well as from private homes. The appearance of private home doors are obviously different to public building doors, which are wider and have more simple frames.

This LaserDoors dataset contains range scans of a SICK S300 LiDAR with an angular resolution of 0.5°. In order to reduce noise in the raw LiDAR data, we averaged 30 scans for each sample. Noise of a certain amount could be added later during data augmentation.

For future applications of the dataset, we additionally recorded color and depth images of a Kinect Azure RGB-D camera, which was placed above the LiDAR at ≈1.2 m height. Although these image data were not used in the present evaluation, each sample comes along with the individual transformations necessary to align the LiDAR to the camera images.

Each door was recorded from both sides at three different recording positions of about 1 m in front of the left door frame, the middle of the door, and the right door frame. For each recording position, the door was in different opening states (closed, unlatched, partly open, and fully open). Finally, 1250 samples were included in the raw dataset.

**Figure 3:**
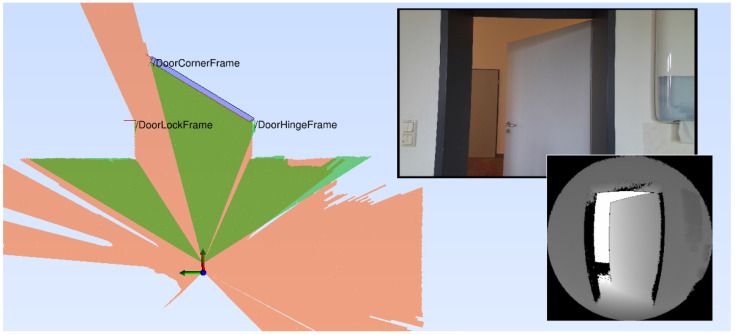
Exemplary sample of the doors dataset consisting of a SICK S300 range scan (orange), Kinect Azure color and depth image, and the annotation of the door’s keypoints. The green range scan is a horizontal line from the depth image. The blue polygon is the model of the door panel with its known geometric parameters.

The data were labeled manually with the position of the hinge, lock, and corner of the blade as well as the metadata for the door database, as described in Section 2. These labels are with respect to the origin of the LiDAR sensor. The horizontal camera position and orientation has been registered to the LiDAR based on a horizontal scan line in the depth image, which could be aligned to the horizontal LiDAR range scan. An example of the LaserDoors dataset is depicted in Figure 3.

We make this dataset available for academic use at the following URL: https://www.tu-ilmenau.de/neurob/data-sets-code/LaserDoors-dataset.

### Augmented Data

The coarsely discretized recording positions of the raw dataset do not reflect the diversity of range scans seen in real applications. To overcome this drawback, an augmentation of recording position has been used for generating training and test data for the machine learning approaches. To that end, the endpoints of the original scan rays were connected to line strips in Cartesian 2D coordinates if the consecutive range values differ less than a threshold of 15 cm. Then, a virtual scan origin is randomly chosen as the original origin plus a random offset of ±20 cm and ±10°. From that virtual scanner position, the scan rays in the typical incremental angular arrangement were intersected with the line strips to yield augmented scan points. During this process, the angular resolution can be modified in order to simulate less accurate hardware. Furthermore, a Gaussian noise can be added to the resulting range values. Additional augmentation has been introduced by flipping the scan direction. In this case, the metadata of the door have to be adapted too.

For training and evaluation of the localization methods, which rely on prior knowledge of the coarse door position in map coordinates, the mapped coordinates of the doors hinge and lock were also augmented. This simulated mislocalization of the robot in the map is chosen in the range of ±10 cm and ±10°.

By means of that augmentation process, the 1250 samples of the raw dataset could be rendered in a diverse training and test dataset of 100 K samples that reflects all possible robot positions that are relevant for door manipulation.

## 5. Methods for Localization of Door Keypoints in Laser Range Scans

For extracting the three keypoints (hinge, lock, and corner) of a door in view, which are necessary to compute all the relevant positions and state information from, we developed and compared several possible approaches. The first method is a heuristic approach exploiting the typical line shape of a door in the scan. For the open states of the door, that line is clearly delimited at the door corner, from where all the necessary coordinates can be calculated. Furthermore, a closed door from the outside is clearly laterally localized by the perpendicular lines of the door frame. The hardest case for a heuristic method is the closed door seen from inside, when it is flush with the wall. This makes lateral localization difficult.

In addition to the heuristic line fitting method, we investigated several machine-learning-based approaches. The problem for these kind of models is that in the raw data, due to the polar-coordinate system, the doors have different appearances depending on the distance to the sensor. In close range, more scan points lie on the door than in far range. Furthermore, the angle of view defines the visible amount of a door.

There are specialized methods that can deal with variable amounts of unordered points. These PointNet [11,12] models take 3D point clouds as input. We modified the network to operate on 2D point clouds and preprocessed the range scans by converting from polar to Cartesian 2D coordinates.

Another popular approach for deep learning models which should operate as a detector in a position-invariant manner are convolutional neural networks. Unfortunately, these approaches need a fixed size input, which is the reason for the necessary preprocessing. In order to normalize the scan pattern with respect to the coarse door position, we re-sampled the scan points by projecting them onto a line going through the coarse door hinge and lock position known from the robot localization in its map. Furthermore, the region of interest is cropped, yielding door-centered histogram features, as described in Section 5.3.1. The goal of the network model, then, is to estimate the offset of the hinge, lock, and corner resulting from the localization error of the robot platform. We implemented a 1D Convolutional Neural Network (CNN) with a Residual Network (ResNet)-like architecture.

The 1D-CNN in first instance is trained in a supervised manner using the labeled lock, hinge, and corner positions as a teacher. This approach encounters a problem when a door’s geometric parameters, which specify the offset of the hinge position to the visible features in the scan line, are not known. In that general case, the model can only learn to predict points on the visible door blade consistently. Without information on the thickness of the door panel, this creates problems when the door is seen from the outside, since the offset of the hinge behind the visible door line is undefined.

To mitigate that problem, we decided to use the given geometric properties of the door (see Section 2) as an additional input, which is fed into the fully connected layers of our network.

Nevertheless, the manually selected keypoints (lock, hinge, and corner) can be suboptimal for a machine learning model. From the domain of 3D object localization, an unsupervised learning method is known, which selects stable, reidentifyable keypoints on its own. This KeypointNet [13] uses two simultaneous views of the same object from different perspectives in order to find visual features that are consistent for different appearances of objects of the same class. Transferred to the door localization domain, this means that in theory, relevant keypoints should be identified that are independent from the actual shape of the door and its frame. Unfortunately, the position of these keypoints with respect to the necessary hinge, lock, and corner are not constant. When such an unsupervised keypoint approach is used, the robot may record door-specific offsets of the keypoints to the labeled poses during mapping. In order to evaluate possible benefits of such an unsupervised approach, we implemented a respective training method for the 1D-CNN model and analyzed the resulting variance of the keypoints.

In the following, the details of the four methods will be given, before the comparative results of an experimental series using the door dataset is discussed.

### 5.1. Heuristic Method

Our first approach, the heuristic method, detects the door’s keypoints by applying a Random Sample Consensus (RANSAC) [14] algorithm to find the door panel and search for predefined features. A similar detection approach using a LiDAR scan to detect the door’s opening angle is described in [7]. This method uses an initial, predefined coarse position combined with consecutive observations to find the exact pivot point of the door and its opening angle. As stated by the authors, the door has to be at least unlatched to be calculated correctly. Furthermore, the door does not need to be localized exactly in 2D space, since the difficult part of manipulating a closed door is excluded from the beginning [7]. Therefore, this method lacks the most important part of the detection by neglecting the lateral localization. With our approach in contrast, we also aimed to localize closed doors from the inside as well as from the outside.

#### 5.1.1. Data Preparation

**Figure 4:**
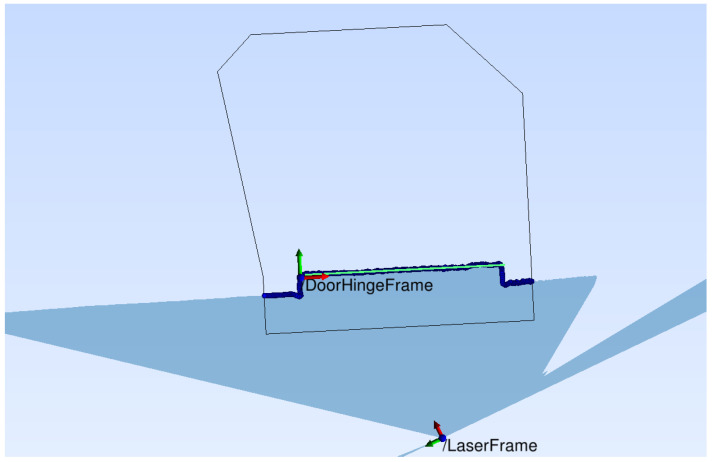
Detection polygon (black) and extracted door line (light green) of a right-sided door.

The data delivered by the LiDAR sensor are represented in polar coordinates having an incrementally growing angle of the scan ray and the respective range readings. For further processing, these polar coordinates are transformed to Cartesian 2D coordinates, yielding a set of scan ray endpoints in a top view of the robot’s environment. From the robot’s localization system, we additionally know the coarse position of the LiDAR in the map and, therefore, the coarse position of the door of interest in the 2D LiDAR coordinates. Thus, a common pre-processing step for all the door localization methods is a crop of a polygonal region of interest from the point set, which is defined with respect to a coordinate system of the door which is aligned to the baseline from hinge to lock of the door of interest. Its size is defined by the door’s width plus 0.3 m along the baseline, and in the opening direction of the door, the size is from −0.35 m to 1.1 times the door width. See Figure 4 for a visualization of that crop region. The polygon adapts to the door width to exclude as many irrelevant points regarding the detection of the door’s keypoints as possible while including all necessary information.

#### 5.1.2. Computation of Door Keypoints

After the preparation of the cropped scan point clouds, a RANSAC algorithm is executed to detect the longest line of points specifying the door’s panel. Afterwards, the keypoints of the lock and hinge need to be calculated. Depending on the observation position of the laser towards the door (inside or outside), different approaches are defined.

If the door is seen from the outside, as shown in Figure 4, the line ends can be calculated from the intersection of the orthogonal lines left and right defined by the door’s frame. To find these shorter lines, again the RANSAC method is applied to a crop of the points that do not belong to the major door line. The intersection points of the extracted lines are used as base points to compute the real hinge and lock keypoints by means of the prior information on the door’s geometry (see Figure 2). This adaptation is necessary since the visible part of the door panel is smaller than the actual panel. As soon as the door is opened further (which can be detected by computing the angle of the door line compared to the hinge to lock baseline in the map), the orthogonal line at the lock side has no meaningful intersection point with the door panel line anymore. Thus, the end point of the door line is used to reverse calculate the hinge position through the door’s width. Conversely, the lock is then computed by searching for a valid depth point at the door’s width in the area of the predefined coarse lock position. The opening angle is defined by the angle of the door line to the new baseline, specified by the detected lock and hinge keypoints.

The second case is a door seen from the inside. This case is difficult in particular when the door is closed, since the depth points of the door’s panel and wall can lie flush with each other depending on the shape of the door’s frame. In experiments trying to detect the door in a lateral manner, we concluded that it is only possible to search for a depth outlier caused by the transition from door panel to door frame and from the frame to the wall. Unfortunately, there is no general threshold that works for all doors. Therefore, an analysis of the point’s perpendicular distances to the line is performed by estimating the variance of these distances. The points within a central area of the door are used to separate noise, induced by various door panels and the sensor noise, from real outliers resulting from the transition to the frame or wall. By iterating the remaining points from center to the lock, we search for the first outlier (≥μ±3σ), while inliers are added to the Gaussian distribution incrementally. Then, the position of the first outlier point is used to calculate the hinge from the door’s known board width inside along the door line and the hinge offset. The detection of the further opened states are reverse calculated from the end point of the door line, similar to the method used for a door seen from the outside, as described above.

#### 5.1.3. Evaluation of the Heuristic Method

**Figure 5:**
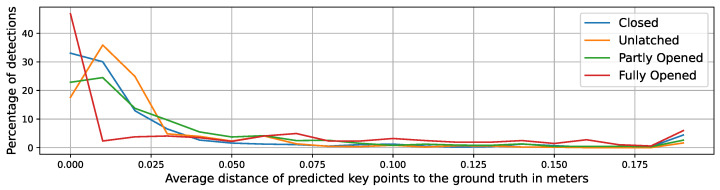
Distribution of position errors (hinge and lock) by the detected door state for both observation sides of the doors on 5000 augmented samples from the LaserDoors dataset.

**Figure 6:**
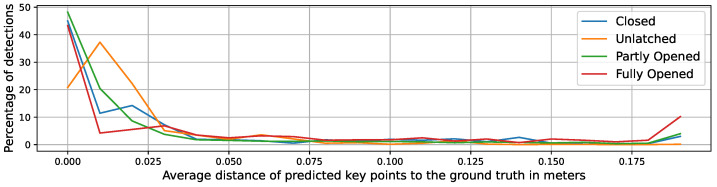
Distribution of position errors (hinge and lock) by the detected door state only for samples from the inside.

The heuristic method, in comparison to the machine learning methods, does not need a training and validation cycle. Thus, we tested the localization method on 5000 augmented samples generated from the LaserDoors dataset, as described above. In Figure 5 and Figure 6, histograms of the average error in steps of 1 cm are given for a dataset containing both views (inside and outside) and on the subset of inside views, respectively. The percentage of high position errors significantly decreases in all four states with an error of around 0.03 m. At the end of the X-axis, all detection errors above the maximum of 0.2 m are summed up, showing a small spike. In particular, for the Fully Opened state, large displacements occur more often (red curve). These errors are induced by missing laser scan points at parts of the door that are nearly parallel to the line of sight. This problem occurs especially with wider opening angles of the door. Then, the end of the door panel is not visible in the scan, causing the end point detection of the line to fail. Since the heuristic approach relies on that end point, it consequently will wrongly predict the keypoints. Fortunately, in these hard-to-detect states of the door, the exact localization of the corner and hinge are of minor relevance, as long as the direction of the line can be estimated correctly, since manipulation of the door handle is not required for pushing the door open.

On average, the localization error, as shown in Table 1, is 0.04 m for arbitrary states of the door. For handling the doors in a closed state, the average localization error of 0.034 m is the more relevant result. This is an improvement by 15–20% in those cases.

The separate analysis of inside views and outside views confirms the described difficulties of lateral localization of doors seen from the inside.

### 5.2. Point Cloud Network

The cropped scan point set after the pre-processing, as described in Section 5.1.1, can alternatively be analyzed using machine learning methods. Although we only have 2D data, the type of input is similar to point clouds that can be generated by depth cameras (3D in that case). For such point clouds, there exist specialized networks which can deal with their characteristics while performing classification and segmentation tasks. The first candidate for such networks was PointNet [15]. It could solve a critical property of point clouds which is the variable number of points contained in the region of interest. Furthermore, the results of a prediction on a set of indistinguishable points need to be invariant against the order of the points in the input, which PointNet realizes by processing each point independently until a final aggregation operation (max pooling) is performed.

Furthermore, affine transformations of the points’ coordinates in the input should not alter the result of a classification. Therefore, the PointNet architecture has a T-Net (transformation network), which estimates a transformation matrix applied to each point in order to bring the point cloud into a normalized position in space.

In [16], the PointNet was used to predict the position of hand keypoints in a 3D point cloud, which shows that regression and localization problems are also solvable by using that network as a feature encoder.

Further improvements of the PointNet, e.g., PointNet++ [12], deal with the recognition of local features rather than considering the whole point cloud as one shape. We do not plan to use the network for classification, but for regression of geometric properties of the point cloud (the coordinates of the hinge, lock, and corner of our door). Thus, the additional invariance properties are not necessary in our case.

**Figure 7:**
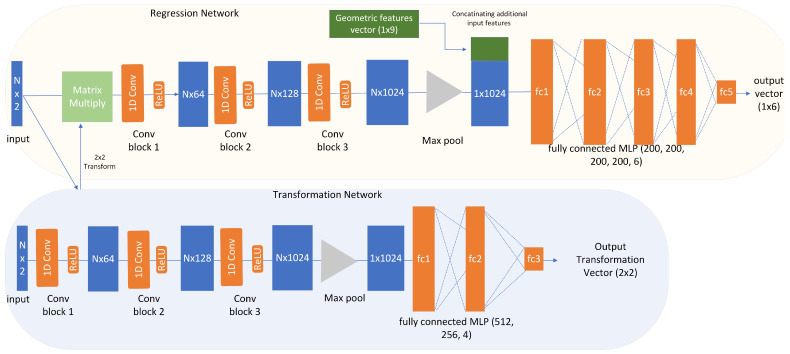
Architecture of the DoorPointNet for 2D point cloud door keypoint localization.

The variant of the network we used is shown in Figure 7. Our DoorPointNet follows the typical PointNet structure, while the transformation network has been changed to predict a 2 × 2 rotation matrix only, rather than the 3 × 3 matrix for 3D points. After the feature transformation by means of three layers of 1D convolution, all point features are merged down by a max pooling operation into a 1024-dimensional feature vector. In order to allow the model to compensate for the invisible properties of the door, the geometric data of the door from the database were concatenated to that 1024-dimensional feature vector of the PointNet encoder.

These additional input features to the network are as follows:

- Opening direction (left or right);
- Side of observation (from inside or from outside);
- Angle of view (LiDAR origin to door baseline from map coordinates);
- Geometry of the door: width, depth inside, depth outside, board width inside, board width outside, and hinge offset (see Figure 2).

A fully connected network then learns the regression of the six dimensional vector containing the X- and Y-coordinates of the desired hinge, lock, and corner of the door.

#### Evaluation of the PointNet Approach

The DoorPointNet has been trained from scratch on a dataset containing 100 K augmented samples of door laser scans having the ground truth position of door lock, hinge, and corner, which are the targets (see Section 4). The dataset also contains the geometric features of the door which have also been used in the network training as additional input features. The loss function of use was the Mean Squared Error (MSE). For enabling batch training, the number of points presented to the network has to be fixed. To that end, we subsampled the point sets from the augmented dataset. If there were less points available, we randomly replicated points from the scan to fill up the input vector. Each point is represented by its X- and Y-coordinates with respect to the cropped regions baseline coordinates.

The dataset has been split into training, validation, and testing at a ratio of 60%/20%/20% at random. The training has been done on an NVIDIA GTX 1080 ti with 128 GB RAM until convergence.

In our experiments, we focused on determining the optimal number of points to be fed into the network, as the number of points used has a direct impact on computational load, and we found that a number of 144 points is sufficient, which reflects the average number of points created by the LiDAR with 0.5° resolution pretty well (see Table 2).

We also conducted experiments by varying the number of samples in the dataset to verify that the dataset is big enough. Table 3 shows the resulting average distance of predicted keypoints (lock, hinge, and corner) to the ground truth.

The remaining error of about 2 cm seems to be inherent to the data, since this limit is also reached for the other approaches.

### 5.3. 1D Convolutional Neural Network

An alternative to the PointNet architecture are simple Feed Forward Neural Networks, also known as Multi Layer Perceptron (MLP). In the form of Convolutional Neural Networks (CNNs), which use shared weights and only local receptive fields to allow deeper and bigger structures, such networks are very successful in the processing of images and other data. Therefore, in our study, we also implemented a 1D convolutional model to solve the door keypoint localization problem. In contrast to the previously discussed DoorPointNet, these models are designed for a fixed size input vector. This makes a manual feature extaction or normalization of the raw LiDAR scans necessary.

#### 5.3.1. Histogram Feature Extraction

**Figure 8:**
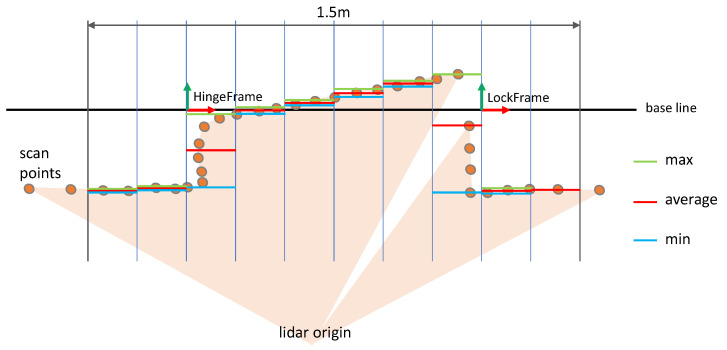
Histogram feature extraction from scan points. In each histogram bin, the min, average, and max vertical positions of the scan points are aggregated.

The point sets from our region of interest (see Section 5.1), therefore, need to be encoded as a fixed size feature vector without losing the generality. Furthermore, we try to compensate for different point densities depending on the observation distance and direction of view. A solution to that problem has been suggested in [17]. They segmented the scan first and extracted histograms of distance values in radial direction. We modified that approach to match our constraints. To that end, the door baseline from lock to hinge in map coordinates is subdivided into a fix number of equidistant intervals distributed over a fixed length of 1.5 m. That 1.5 m line is symmetrically centered at the supposed door center in map coordinates, which is the middle of the hinge and lock. Figure 8 shows that subdivision. By means of that, enough context is included to also find the wall when the door is fully open and the robot is mislocalized by 0.2 m. All scan endpoints in our region-of-interest crop are then projected perpendicular onto the baseline. Then, the vertical positions of the points are averaged for each of the intervals. Additionally, the minimum and maximum Y-positions are determined for each interval yielding a feature vector of three times the number of intervals. Comparative studies showed that 150 × 3 is optimal given the angular resolution of our dataset (0.5°).

#### 5.3.2. Supervised Training for 1D Convolutional Neural Network

**Figure 9:**
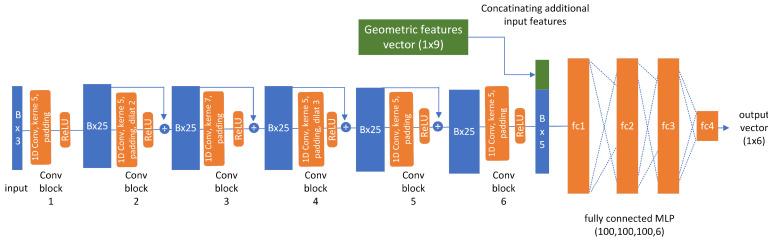
Architecture of our 1D-CNN for processing histogram features of door range scans into predictions of X- and Y-coordinates of the three keypoints of a door.

Using the normalized histogram features extracted from the point sets, an analysis with a 1D Convolutional Neural Network (1D-CNN) is possible. To that end, we implemented a network that uses dilated convolutions in six layers. The architecture search yielded an optimal number of 25 features for the intermediate layers, and the layers were padded such that their dimension stay constant at the number of bins in the histogram features. The first layer has three features, which comprise the average, min, and max distance of the points to the baseline as given in the histogram features. In between the convolution layers, ReLU activation is performed, and the results are added to the previous layer’s input similar to a Residual Network. After the convolution layers, the resulting feature vectors were flattened, and the geometric properties of the door (similar to Section 5.2) are concatenated to the feature vector as auxiliary input. Then, three fully connected layers with ReLU, each having 100 neurons, follow. The output layer has linear activation and comprises the six neurons for X- and Y-coordinates of the door’s hinge, lock, and corner frame. These target coordinates were given in the baseline coordinate system, because the histogram feature extraction cancels out any information on absolute positions in LiDAR coordinates. Coordinates later can be transformed to robot or laser coordinates with the known origin of the door baseline. Figure 9 illustrates the architecture of the described network.

The 1D-CNN in the following is used in two different ways. The first one is a supervised training. Here, the simple MSE to the ground truth labels are used as a loss function. The second form of application for the 1D-CNN is in an unsupervised training scheme, as described in Section 5.3.4.

#### 5.3.3. Evaluation of the 1D-CNN Supervised Training Approach

The model is trained with the ADAMw optimizer until convergence (200 epochs). Since the network is so lightweight, the training could be performed on a CPU (Intel Core i5, 8th Gen with 8 GB RAM), taking 33 s per epoch. Validation loss seems not to increase at the end of the training, which shows that there is a lot of uncertainty in the data, and the network is not too big. We conducted an architecture search, trying different numbers of hidden and convolutional layers, which ensures that the presented configuration is the best performing.

**Figure 10:**
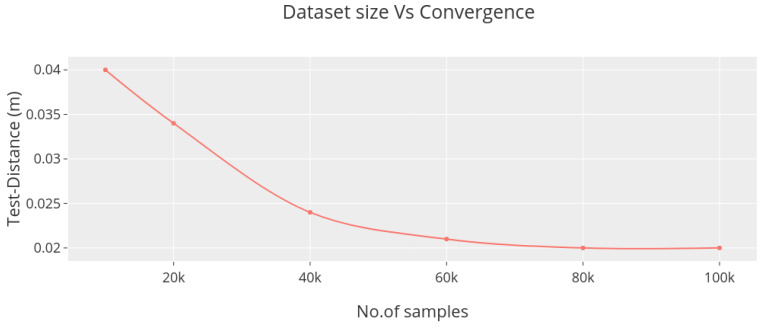
Achievable accuracy of the 1D-CNN for different dataset sizes.

Similar to the experiments with the DoorPointNet, we trained the 1D-CNN on differently sized subsets of the 100 K augmented dataset again using the 60%/20%/20% split. The results shown in Figure 10 prove that the complexity of the network fits the complexity of the 100 K samples dataset. Further increasing the amount of the training data seems not to provide any improvements.

The resulting average absolute position error of the predicted keypoints (2 cm) is comparable to the results of the DoorPointNet approach, which indicates that this might be a limit that is related to the dataset’s inherent uncertainty rather than being caused by deficits of the used model.

#### 5.3.4. Unsupervised Keypoint Learning

The manually labeled keypoints of the door (hinge, lock, and corner) are partly dependent on not-observable properties of the door. Therefore, the dataset seems to be partly contradictory and has a high level of uncertainty, which cannot be mastered by any supervised learning method.

Therefore, in the image processing domain, unsupervised methods have been suggested in the literature, e.g., the KeypointNet [13]. This framework tries to find the best-suitable keypoint positions at the objects of interest on its own without any human label input. Only the number of keypoints to be extracted have to be defined. KeypointNet comprises a set of loss functions that operate on the predicted keypoints in two different views of the same object. Since the transformation between these two views is known, the keypoints found in one image can be projected onto the image plane of the other view and the deviation yields a loss. By means of that, a robustly identifyable structure of the object in the images is selected automatically. Additionally, the 3D coordinates of the keypoints can be reconstructed from the two views and the network learns to predict the approximate depth from monocular images.

This idea of unsupervised keypoint selection can also be ported for the processing of 2D laser range scans with the opportunity to overcome the problem of suboptimal label positions. To that end, we adopted the training with two different views of the same object and developed an alternative loss function for the 1D-CNN.

The unsupervised training aims to predict two keypoints in 2D coordinates of the feature vector’s baseline (similar to the X- and Y-coordinates of the predicted lock, hinge, and corner of the supervised approach), which in first place do not have a given semantics, but which should be fixed to the geometric features of the door. These two predicted points are sufficient to define a rigid transformation (translation and rotation only) that brings the input scan into a normalized position with respect to the actual door. Once the door is localized that way, the exact coordinates of the lock and hinge should be fixed for each door and can be stored in the database during mapping of the robot’s operational environment.

**Figure 11:**
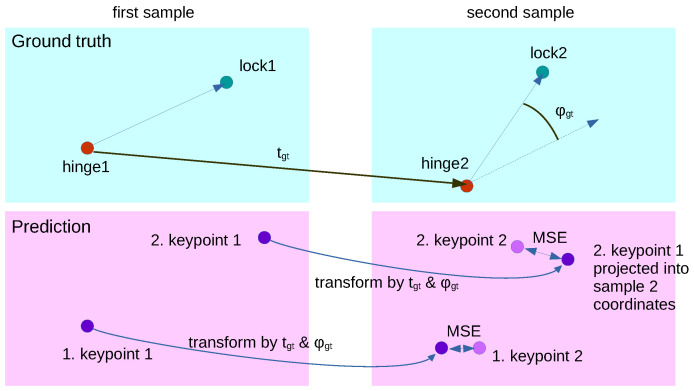
Function of the Consistency loss using ground truth and prediction of two samples of the same door.

The training process operates on two different samples of the same door simultaneously. For both samples, the ground truth positions of the doors are known and from these, the transformation consisting of translation $t_{gt}$ and rotation $\phi_{gt}$ can be computed (see Figure 11). Consistent keypoint positions are now found by the network if the projection of the keypoints from sample one into the domain of sample two are close to the positions of the keypoints predicted in sample two. To that end, the Consistency loss function does exactly this projection and evaluates MSE of the keypoint coordinates, as shown in Figure 11. This process is also performed the other way around.

Training with the Consistency loss alone is not successful, since keypoints can converge to a singular point. That makes it impossible to compute the transformation correctly. Thus, a further loss term is needed which punishes the network if the predicted keypoints $k_{1}$ and $k_{2}$ come to close to each other. This *distance loss* is defined as follows:

(1) $$L_{dist}=ReLU\left(-|k_{1}-k_{2}|\;+\;0.8\right)$$

where the 0.8 has been selected to match the average door width, but the exact value is of minor importance.

#### 5.3.5. Evaluation of the Unsupervised Training Method for the 1D-CNN

Since the network does not predict the absolute position of the doors’ points of interest, we cannot compute a position error for the lock, hinge, and corner. However, we analyzed the stability of the predicted keypoint’s position in door coordinates for the individual instances of the doors in the dataset. Regardless of the absolute relative position of the keypoints to the door, the variance is comparable to the mean squared error of the lock and hinge position predicted with the other approaches.

Table 4 shows the results for different splits of the original dataset. For the outside views over all opening states, the deviation of the predicted keypoints is in the range of 3 cm, which is slightly worse than the results of the supervised approaches. For the inside views, the lateral localization is even worse: about 5 cm on average, which reflects the difficult situations of closed doors which are flush to the wall. The focus on only closed doors in a separate training confirms that assumption as shown in the last rows of the table. Here, the lateral deviation (y-standard deviation) is even bigger than the average of all opening states.

Considering the absolute values of the deviation vectors, the overall accuracy of inside and outside views is 0.05 m. In conclusion, the unsupervised method basically worked as expected and yielded some stable keypoints in the door scans, but it could not outperform the supervised training with the manually labeled door keypoints (lock, hinge, and corner).

## 6. Discussion

Table 5 summarizes the results of the four different approaches to the door localization problem.

The similar results of two different machine learning models give rise to the assumption that these 2 cm are a limit inherent to the dataset. On the other hand, the supremacy of machine learning approaches over heuristic strategies also showed up in the evaluation results. The heuristic line fitting approach showed significantly worse position errors for the evaluation on the generic dataset (4 cm in contrast to 2 cm). Nevertheless, for practical realization of a door-opening robot, the heuristic approach proved to be sufficient. We realized an application for autonomous transport tasks including door traversal on two different robot platforms. One is a TIAGo robot from Pal Robotics equipped with a SICK TIM571 LiDAR, having an angular resolution of 0.33°. With this sensor, the heuristic method could be tuned to recognize doors in closed state that are completely flush to the wall. These doors’ frames show only a 4 mm gap between frame and board as well as between the frame and the wall. The machine learning models in this particular case fail due to the lack of training data. The reason for that is that the dataset has been recorded with a SICK S300 LiDAR, which had only a 0.5° resolution. The second robot we used is a SCITOS G5 by MetraLabs GmbH equipped with a SICK S300 as well. As already mentioned, this sensor is not able to resolve the relevant features of our particular doors. The solution we found shows the benefit of the heuristic approach. Since the robot is equipped with a Kinect Azure depth camera as well, we could extract a virtual LiDAR scan from the depth image of that camera. This has an angular resolution of less than 0.1° and, therefore, could be used with the heuristic method without any new training. The localization worked, although the depth image suffers from different artifacts related to the materials of the door and walls. The remaining localization errors could be handled by the manipulation strategy of the robot, which incorporates force feedback in order to compensate the lack of perception of the exact handle position.

The machine learning models on the other side would have required preparation of specialized training data for that particular door and sensor configuration. With the comparison on the dataset in mind, the application of an adapted neural network approach is promising in order to reduce the localization error of the door further.

The problems of the machine learning models with the unseen door shows that the dataset is not yet big enough to train a general-purpose door detector able to also generalize for new situations. The dataset, in fact, has only been recorded to allow an objective direct comparison of the methods.

In a live processing implementation of the 1D-CNN on the robot, the detection showed a sensitivity to the sensor noise. In contrast to the heuristic method, the predicted hinge and lock position jitter over time. By means of a geometric median filter, we were able to stabilize the outputs of the 1D-CNN such that the manipulation and grasp pose planning could operate smoothly.

A further finding of the experiments was that the unsupervised keypoint detection model is not yet optimal. We will further investigate that approach by changing the output encoding of the network, which could predict parameters of a transformation directly rather than predicting keypoints. Furthermore, the selection of the pairs of examples in the training process might influence the performance and will be researched further. Nevertheless, we could show that the unsupervised keypoint learning approach [13] also works for 2D LiDAR data. Therefore, it could also be used for other detection and localization problems.

In terms of efficiency, all the presented methods are pretty lightweight. The data stream from our LiDAR sensors can easily be processed in real time on a CPU only.

Among the machine learning methods, the DoorPointNet has the advantage of not requiring a manual feature extraction. Rather, it consumes the raw point sets and may be able to find better features for the final prediction. On the other hand, the presented feature extraction method for normalizing the LiDAR point cloud could also help to analyze other targets by means of neural networks. The person detection problem, which had been solved by means of a boosting approach using support vector machines [17], could be improved by using CNNs instead.

## 7. Conclusions

In this article, we presented a couple of new approaches to the problem of localizing doors in LiDAR scans. In the literature, there is no quantitative analysis of similar methods so far; thus, we recorded and offered a manually labeled dataset for the door keypoint localization problem.

In evaluations, we found that the laser-based detection of the door’s keypoints is an efficient and reliable alternative to image-based detection approaches. The machine learning methods could beat the line-fitting heuristics by halving the error. Among the supervised trained networks, there is no big difference in the remaining position error, i.e., about 2 cm. This seems to be a limit that is caused by the LiDAR resolution and the diversity of doors’ appearance in LiDAR scans.

However, the exact selection of the method depends on external circumstances, e.g., availability of training data and the required generalization capabilities of the robot in its operational environment. Even the heuristic method, which performed worse than the networks, was also successfully used in a practical application to open doors.

## Figures and Tables

**Table 1:** Average distance between predicted keypoints and ground truth using the heuristic approach.

Side of View	State of the Door	Distance (m)
both	all	0.0401
inside	all	0.0417
outside	all	0.0386
both	closed, unlatched	**0.0340**

**Table 2:** DoorPointNet training experiments with different no. of input points on 100 K dataset.

**Input Points**	144	240	720
**Test-Distance (m)**	0.020	0.020	0.021

**Table 3:** DoorPointNet training experiments with different length of dataset with 144 no. of scan points as input, the network is trained for 200 epochs.

**Dataset Size**	100,000	60,000	20,000
**Test-Distance (m)**	0.02	0.023	0.026

**Table 4:** Stability of keypoints from the unsupervised training of 1D-CNN evaluated in door-specific ground truth coordinates (x—perpendicular to door baseline, y—parallel to baseline).

Dataset	1. Keypoint	2. Keypoint
	Standard Deviation [m]	Standard Deviation [m]
	(x,y) abs	(x,y) abs
outside all states	(0.025, 0.022) 0.033	(0.028, 0.035) 0.044
inside all states	(0.027, 0.044) 0.051	(0.036, 0.063) 0.072
outside only closed	(0.024, 0.026) 0.035	(0.032, 0.036) 0.048
inside only close	(0.035, 0.052) 0.062	(0.052, 0.073) 0.089

**Table 5:** Average distances of predicted door keypoints for the different methods evaluated on the augmented LaserDoor dataset.

Method	Distance [m]
Heurisic Line Fitting	0.04
DoorPointNet	0.02
1D-CNN supervised	0.02
1D-CNN unsupervised	0.05

## Data Availability

We provide access to our dataset of door scans for academic use: https://www.tu-ilmenau.de/neurob/data-sets-code/LaserDoors-dataset.
